# Potential Use of Silica Nanoparticles for the Microbial Stabilisation of Wine: An In Vitro Study Using *Oenococcus oeni* as a Model

**DOI:** 10.3390/foods9091338

**Published:** 2020-09-22

**Authors:** Kamila Pachnowska, Krzysztof Cendrowski, Xymena Stachurska, Paweł Nawrotek, Adrian Augustyniak, Ewa Mijowska

**Affiliations:** 1 Department of Nanomaterials Physicochemistry, Institute of Chemical and Environment Engineering, West Pomeranian University of Technology in Szczecin, Piastów Avenue 45, 70-311 Szczecin, Poland; kamila.mijowska@zut.edu.pl (K.P.); krzysztof.cendrowski@zut.edu.pl (K.C.); ewa.mijowska@zut.edu.pl (E.M.); 2 Department of Microbiology and Biotechnology, Faculty of Biotechnology and Animal Husbandry, West Pomeranian University of Technology, Szczecin, Piastów Avenue 45, 70-311 Szczecin, Poland; pawel.nawrotek@zut.edu.pl; 3 Department of Chemical and Process Engineering, Faculty of Chemical Technology and Engineering, West Pomeranian University of Technology, Szczecin, Piastów Avenue 42, 71-065 Szczecin, Poland; adrian.augustyniak@zut.edu.pl; 4 Chair of Building Materials and Construction Chemistry, Technische Universität Berlin, Gustav-Meyer-Allee 25, 13355 Berlin, Germany

**Keywords:** silica nanospheres, antibacterial agents, stirring, *Oenococcus oeni*, process stabilisation

## Abstract

The emerging trend towards the reduction of SO_2_ in winemaking has created a need to look for alternative methods to ensure the protection of wine against the growth of undesired species of microorganisms and to safely remove wine microorganisms. This study describes the possible application of silica nanospheres as a wine stabilisation agent, with *Oenococcus oeni* (DSM7008) as a model strain. The experiment was conducted firstly on model solutions of phosphate-buffered saline and 1% glucose. Their neutralising effect was tested under stirring with the addition of SiO_2_ (0.1, 0.25, and 0.5 mg/mL). Overall, the highest concentration of nanospheres under continuous stirring resulted in the greatest decrease in cell counts. Transmission electron microscope (TEM) and scanning electron microscopy (SEM) analyses showed extensive damage to the bacterial cells after stirring with silica nanomaterials. Then, the neutralising effect of 0.5 mg/mL SiO_2_ was tested in young red wine under stirring, where cell counts were reduced by over 50%. The obtained results suggest that silica nanospheres can serve as an alternative way to reduce or substitute the use of sulphur dioxide in the microbial stabilisation of wine. In addition, further aspects of following investigations should focus on the protection against enzymatic and chemical oxidation of wine.

## 1. Introduction

Winemaking (vinification), i.e., techniques of transforming grape must into wine, has to guarantee its stability in such a way that does not affect its organoleptic properties [1]. Currently, sulphur dioxide (SO_2_) is widely used in wine stabilisation, to ensure protection against enzymatic and chemical oxidation and the growth of undesired species of microorganisms [2,3,4,5]. However, in the last two decades, more and more reports indicated that SO_2_-derived compounds had caused many adverse clinical effects on human health. Therefore, the legislative rules, health consciousness of consumers, and the emergence of organic production have resulted in a general trend towards the reduction of SO_2_ amount in food [2,6]. Consequently, researchers are looking for alternative methods allowing the reduction or even elimination of SO_2_ as a preservative, without significantly changing the quality attributes of wine [2,3,6,7,8,9]. Research papers have indicated many varied methods, including the use of biological (e.g., microbial resources [10,11]) and physical methods (e.g., pulsed electric fields [12,13], high pressure [14,15,16], ultraviolet radiation (254 nm) [17,18]), electron beam irradiation [19], or additives to the musts or wines, such as dimethyl dicarbonate [20,21], bacteriocins [22], chitosan [23], chitooligosaccharide [24], lysozyme [25,26,27], phenolic compounds [27,28] or α-pinene terpene [29]. The above-mentioned methods are biocompatible and have promising properties in reducing SO_2_ content. However, their limitations, such as high costs and adverse influence on sensory properties of wine, prevent them from successfully replacing the sulphuring process. Therefore, further investigations are necessary to improve or combine existing techniques, or to develop new ones.

The use of nanomaterials as antibacterial and antifungal agents is mainly concentrated around the nanostructures of silver [30], copper [31], and nanoparticles with photocatalytic properties, such as titanium dioxide [32]. A study on the synthesis of silica spheres was first documented in the sixties by Warner Stöber [33]. Since 1968, methods for the synthesis and the potential use of silica nanomaterials have been extensively studied. Methods for obtaining the solid and porous silica nanostructures of spherical [34] and cylindrical shapes [35], in the form of flakes [36], as well as nanometric layer/coatings [37] have been developed. These methods allow us not only to synthesize a wide spectrum of differently shaped structures, but also to gain materials with a strictly defined size [38]. Silica nanomaterials are characterised by high thermal and chemical stability and high biocompatibility, as confirmed by in vitro and in vivo studies [39,40]. The chemical, physical, and biological properties of silica nanomaterials have allowed the development of a wide range of applications for them as additives to cement composites [41], drug carriers (antibacterial, anticancer) [42], carriers of metals and metal oxides for their potential use in medicine [43] or catalysis [44], and in diagnostics for the separation of DNA [45] or templates for synthesis of other nanomaterials [46]. Silica nanostructures also show a tendency for biodegradation after exposure to living organisms and in environments imitating conditions prevailing in living organisms. Therefore, the application of silica nanomaterials as carriers for the transport of silver, copper, and titanium dioxide as antibacterial agents has been proposed [47,48].

Preliminary studies on the biocompatibility of silica nanostructures (nanospheres, nanotubes, nanoflakes) showed no cytotoxic properties [40,43]. Our study on L929 mouse fibroblast cells showed a cellular uptake that was dependent on the size of nanosphere. Confocal microscope images showed that nanospheres were localised around the cell nucleus after 24 h of incubation. Cellular aggregations and preferential accumulation around the nucleus were also observed, although nanomaterial did not cause any cytopathic effects. Studies using a confocal microscope confirmed that the silica nanostructures are biocompatible even when internalised by L929 cells [40,43]. Our research on the biocompatibility of silica nanoplates confirmed previous results regarding silica nanospheres and nanotubes [49].

In winemaking, it is also important which microorganisms perform the fermentation processes. The management of these biological resources, associated with alcoholic fermentation, have an impact on the later spontaneous malolactic consortium, by generating different taxonomic composition of the bacterial communities, and modulating malolactic fermentation performance. Therefore, changes in the vinification environment can shape the diversity of malolactic consortia [50]. However, these consortia also contain microbes responsible for wine spoilage that produce undesirable compounds, such as biogenic amines [51,52]. *Oenococcus oeni*, a species well-adapted to the harsh adverse conditions of the wine environment that is able to perform the malolactic fermentation [53], is also the most desirable bacterium in winemaking. However, it can be co-responsible for the wine spoilage [54]. Thus, the development of methods providing the efficient control of this bacterium could allow to obtain the product that maintains a desirable quality. Therefore, *Oenococcus oeni* (DSM7008) commercial strain was used as a model microorganism in our study, investigating the use of silica nanostructures (SiO_2_) without any additional antibacterial/antifungal agents as an innovative, biocompatible, and biodegradable agent towards the reduction of bacteria from selected suspensions.

## 2. Materials and Methods

### 2.1. Materials

Silica precursor (tetraethyl orthosilicate, or TEOS) was bought from Sigma Aldrich. Ammonium solution and ethanol were provided from Chempure (Piekary Śląskie, Poland). Silica nanospheres were synthesised by hydrolysis of the silicates (TEOS), according to the method published previously [46]. Briefly, ethanol (100 mL), and ammonia solution (5 mL) were mixed together and placed under reflux (55 °C) with contentious stirring. After stabilisation of the temperature, TEOS (3 mL) was added and stirred for 24 h. The obtained product was dried in air.

A commercial strain of *Oenoccocus oeni* (DSM7008) VINIFLORA OENOS (CHR HANSEN) was used in this study as a model microorganism. The bacterium was a part of the Collection of Department of Microbiology and Biotechnology, at West Pomeranian University of Technology, Szczecin.

### 2.2. Preparation of Oenococcus oeni Culture

Bacterium was stored in de Mann, Rogosa, and Sharpe broth (MRS; BioMaxima, Lublin, Poland) with 20% (*v/v*) glycerol at −20 °C prior to experiments. The strain was recovered by streaking the defrosted suspension directly onto MRS agar (BioMaxima, Lublin, Poland) plates and incubated at the room temperature for 48 h. Afterwards, a single colony was used to inoculate 50 mL of MRS broth. Bacterial cells were incubated at room temperature with shaking (110 rpm) in an orbital rotating shaker (Shaker-Incubator ES-20, BioSan, Józefów, Poland) for 48 h. Then bacterial suspension was used for further experiments.

### 2.3. Reduction of Oenococcus oeni Numbers in PBS and Glucose by Silica Nanospheres with Stirring

Microorganisms’ neutralisation was carried out in sealed glass reactors equipped with magnetic stirrers. Phosphate-buffered saline (PBS; 135 mM NaCl, 1.3 mM KCl, 0.5 mM KH_2_PO_4_, and 3.2 mM Na_2_HPO_4_; pH 7.4) and 1% (*w/v*) glucose solution were used as experimental media. *O. oeni* (DSM7008) overnight culture suspension was brought to 1.0 MF (McFarland standard), and 2 mL was added to the 50 mL of PBS and 1% glucose. Nanomaterials were added to the liquid contaminated with bacteria in the form of a concentrated suspension. Prior to the study, nanomaterial was dispersed in 5% of the studied volume with ultrasounds. The nanomaterial was studied in the final concentrations of 0.1, 0.25, and 0.5 mg/mL, respectively. The suspensions were continuously stirred at room temperature (~22 °C), with the constant speed of 500 rpm for 90 min. The bacteria-reducing properties of the silica nanomaterial were analysed during the stirring with seven measuring points, each after 15 min, by collecting 1 mL of the suspension, and immediately diluting and plating on MRS agar. Samples were then incubated at room temperature for 48 h, and the grown colonies were counted. All measurements were conducted in triplicate and in three independent experiments.

### 2.4. Reduction of O. oeni Counts by Silica Nanospheres without Stirring

The silica nanospheres’ short cytotoxicity test on the *O. oeni* (DSM7008) cells was conducted in terms of the influence of different media (studied in the Section 2.3) on the level of nanomaterial cytotoxicity without stirring. Overnight culture of *O. oeni* (DSM7008) was brought to 1.0 MF by diluting in MRS broth, and 2 mL was added to the 50 mL of PBS and 1% glucose. Silica nanospheres were added into the mixtures to reach the final concentration of 0.5 mg/mL, which was the highest nanomaterial concentration tested. Samples were then incubated at room temperature for 3 h without shaking, and were then collected immediately, diluted, and plated on MRS agar. Plates were then incubated at room temperature for 48 h, and the grown colonies were counted. The test was conducted in triplicate in two independent experiments in PBS and 1% glucose solution.

### 2.5. Reduction of Oenococcus oeni Numbers in Wine by Silica Nanospheres with Stirring

The experiment was carried out in the sealed glass reactors equipped with magnetic stirrers, as in Section 2.3. As the experimental medium, young wine was used, which was produced from grapes of the red ‘Regent’ cultivar, grown at a research station of the West Pomeranian University of Technology, Szczecin (Poland). After the end of alcoholic fermentation, young wine was collected, cleared by filtration (PC filters, 0.45 nm) and used in the experiments. *O. oeni* (DSM7008) overnight culture suspension was brought to 1.0 MF (McFarland standard) and 2 mL was added to 50 mL of the cleared young wine. Nanomaterials were added to the suspension in the concentrated form. Prior to the study, the nanomaterial was dispersed in 5% of the studied volume with ultrasounds. The nanomaterial was studied in the highest concentration of 0.5 mg/mL. The suspensions of the tested and control samples were continuously stirred at room temperature (~22 °C), with the constant speed (500 rpm) for 90 min. Bacterial counts were determined during the stirring with samples (1 mL each) taken at seven measuring points (with 15 min interval), immediately diluted, and plated on MRS agar. Samples were then incubated at room temperature for 48 h, and the grown colonies were counted. All measurements were conducted in triplicate.

### 2.6. Silica Nanospheres Biodegradation Studies

Degradation studies of the silica nanomaterials were carried out in both selected media (see Section 2.3). The shape and size of silica nanospheres were analysed. The nanostructures were added into the glucose and PBS solutions, in order to obtain concentrations up to 0.5 mg/mL, as in previous tests. Samples for the microscopic analysis were collected after 1 h of incubation at the room temperature. The initial nanostructures were compared to silica nanospheres obtained after the incubation.

### 2.7. Visualization and Physiochemical Characteristics of the Silica Nanospheres

Thermogravimetric analysis (TGA) was conducted under an argon flow with a heating rate of 10 °C min^−1^, using a TA Instrument SDT Q600. The morphology of the samples was examined with a transmission electron microscope (TEM; Tecnai G2 F20 S-TWIN, FEI) equipped with a high-angle, annular, dark-field HAADF detector (STEM), a module X-ray energy dispersive spectroscope (EDX), and scanning electron microscopy (SEM; TESCAN, VEGA SBU3).

### 2.8. Statistical Analysis

One-way ANOVA was used to statistically analyse the results, along with Tukey’s post-hoc test. Results were examined on three levels (*p* < 0.05, 0.01, or 0.001). The assumptions for the ANOVA were tested for each dataset.

## 3. Results

### 3.1. Silica Nanosphere Characteristics

**Figure 1:**
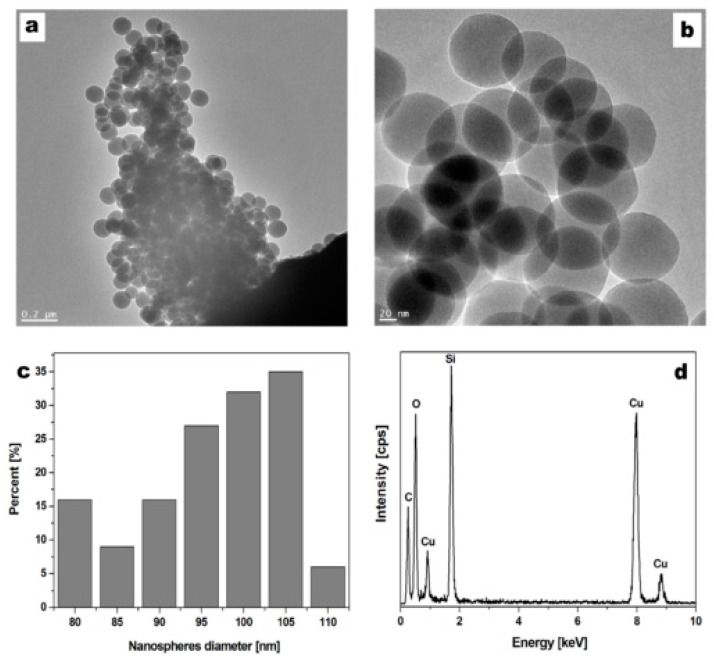
Transmission electron microscope (TEM) images (**a**,**b**), diameter size distribution (**c**), and X-ray energy dispersive spectroscope (EDX) spectrum (**d**) of the silica nanospheres.

Figure 1a,b presents the TEM images of the synthesised silica nanospheres, with a diameter of approximately 100 nm. The obtained size distribution was based on the analysis of TEM images (Figure 1c) of the nanospheres. The elemental composition of the nanostructure determined by EDX spectroscopy (Figure 1d) indicates that the nanospheres were composed of silicon and oxygen. Peaks assigned to the carbon and copper came from TEM grid. The TGA of the silica nanospheres is presented in the Supplementary Information (Supplementary Materials, Figure S1). According to the TGA, the silica nanospheres contained around 3% of organic impurities.

### 3.2. Reduction in Oenococcus oeni Counts by Silica Nanospheres with Stirring

**Figure 2:**
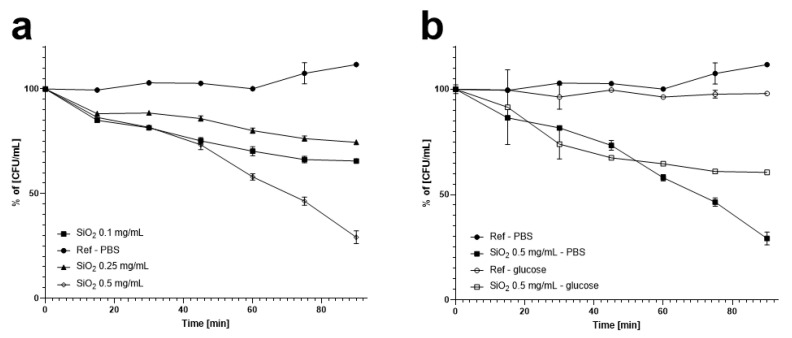
Diagrams of the *Oenococcus oeni* (DSM7008) viability after bacteria exposure to the silica nanospheres (SiO_2_) in phosphate-buffered saline (PBS) solution (**a**) and glucose (**b**) under stirring.

Figure 2 presents the changes in colony counts under 90 min-long stirring with and without the addition of the silica nanomaterials, in terms of initial microbial counts. The studies showed a positive relationship between the nanomaterial concentration and *O. oeni* (DSM7008) counts in the PBS solution (Figure 2a). Moreover, it was recorded that microorganism viability of the control sample was not greatly affected by stirring. After 60 min of incubation with continuous stirring, the silica nanostructure concentrations of 0.1 mg/mL, 0.25 mg/mL, and 0.5 mg/mL caused a decrease in the bacterial counts by 30%, 20%, and 42%, respectively, in comparison to the control. However, after 90 min of the experiment, this parameter was further decreased to 46%, 37%, and 82%, respectively (Figure 2a). Overall, the highest concentration of nanospheres combined with continuous stirring resulted in the largest decrease in the colony counts. Therefore, the nanomaterial concentration of 0.5 mg/mL proved to show the highest neutralising activity, causing a significant reduction of the *O. oeni* (DSM7008) live cells. For that reason, it was selected to assess the effect of the used medium on its neutralising activity (Figure 2b). Compared to the test conducted in PBS buffer, with 0.5 mg/mL of silica nanospheres with continuous stirring, where the viable cell count was reduced by 42% after 60 min and 82% after 90 min, the change of the media for a glucose solution reduced the number of colonies by 32% and 38% after 60 min and 90 min, respectively (Figure 2b).

### 3.3. Reduction in O. oeni Counts by Silica Nanospheres without Stirring

**Figure 3:**
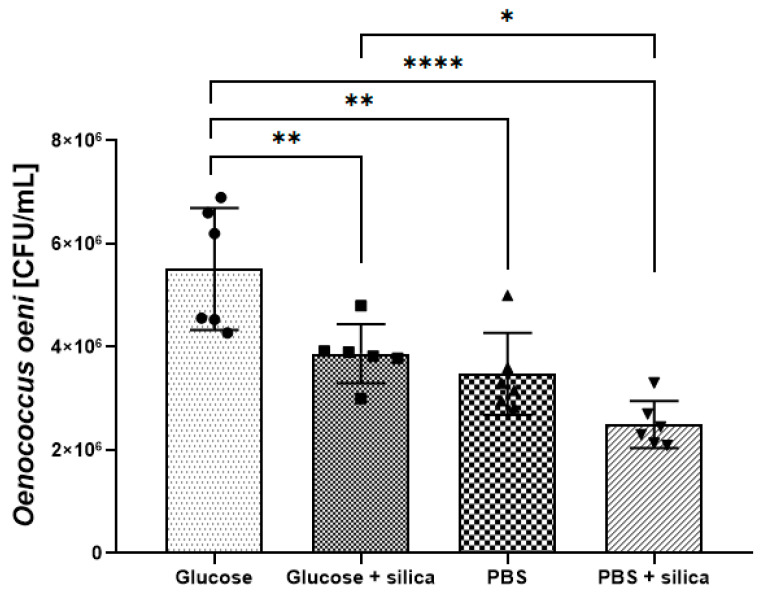
Diagram of silica nanospheres’ cytotoxicity effect on *Oenococcus oeni* (DSM7008) cells in PBS and glucose solutions. Data are expressed as mean ± SD. Asterisks within a bar graph indicate significant differences between the means; * *p* < 0.05, ** *p* ≤ 0.01, **** *p* < 0.001, one-way ANOVA followed by the Tukey’s multiple-comparison test.

The changes in colony counts after 3 h incubation with and without the addition of the silica nanomaterial and without stirring under different media conditions are presented in Figure 3. The studies showed statistically significant differences between the used media (PBS or glucose solution), nanomaterial presence, and *O. oeni* (DSM7008) counts. After incubation in the glucose solution, 5.51 × 10^6^ CFU/mL were reached, while with the presence of silica nanospheres, a significant decrease (*p* ≤ 0.01) was recorded at 3.87 × 10^6^ CFU/mL. Incubation in PBS resulted in colony counts at the level of 3.47 × 10^6^ CFU/mL, while with the addition of the nanomaterial the number of colonies decreased to 2.49 × 10^6^ CFU/mL. Therefore, the addition of the nanomaterial reduced the number of colony-forming units, irrespectively of the medium type, although with a statistically significant difference when the glucose solution was used. However, when comparing the influence of the used media on the samples treated with silica nanoparticles, there was a statistically significant decrease (*p* < 0.05) in the microbial count between the glucose solution (3.87 × 10^6^ CFU/mL) and PBS (2.49 × 10^6^ CFU/mL) (Figure 3).

### 3.4. Reduction of Oenococcus oeni Numbers in Wine by Silica Nanospheres with Stirring

**Figure 4:**
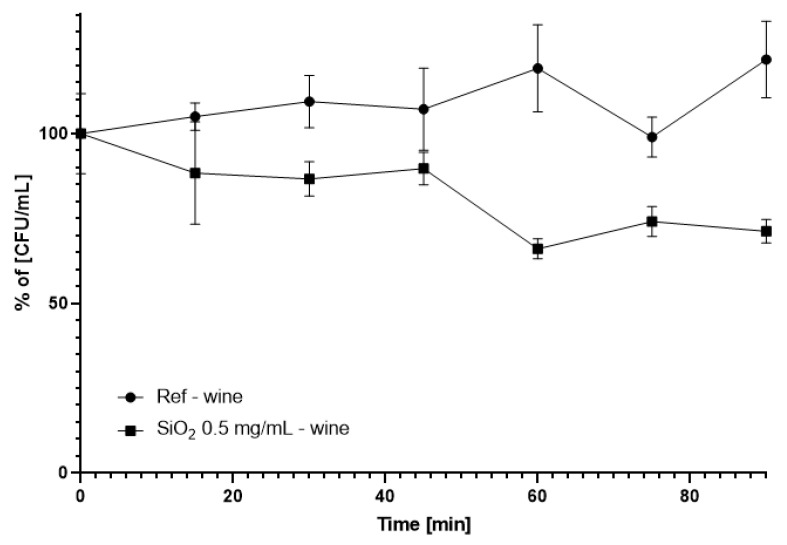
Reduction of the commercial *Oenococcus oeni* (DSM7008) strain in wine after exposure to the silica nanospheres (SiO_2_) under continuous stirring.

The outcome of experiments presented in Section 3.2. and Section 3.3. allowed us to plan and execute further tests in young red wine, with the use of the highest studied concentration of silica nanospheres (0.5 mg/mL). The results indicated a decrease in the bacterial counts by 53% after just 60 min of incubation with continuous stirring, compared to the control. After 90 min of the experiment, this parameter remained at a similar level (reduced by ca. 51%) (Figure 4).

### 3.5. Microscopic Visualisation

**Figure 5:**
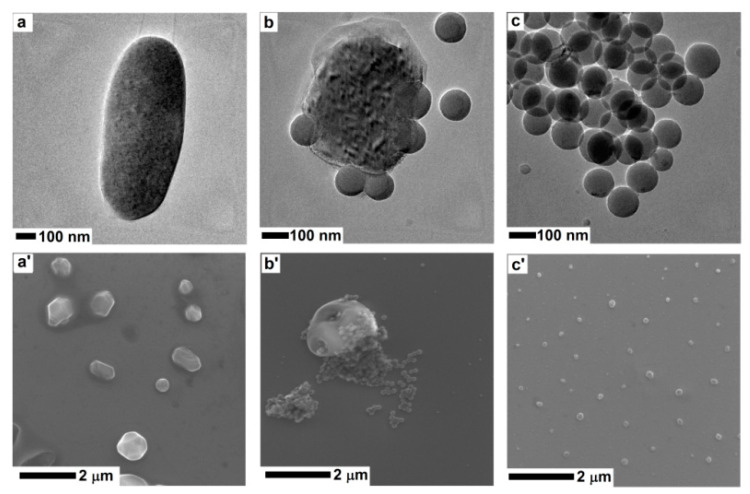
TEM and SEM (scanning electron microscope) images of *Oenococcus oeni* (DSM7008) (**a**,**a’**), *Oenococcus oeni* (DSM7008) stirred with nanospheres (**b**,**b’**), and pristine silica nanospheres (**c**,**c’**), all from PBS solution.

The TEM and SEM analysis of the silica nanospheres incubated with Oenococcus oeni (DSM7008) cells are presented in Figure 5. All presented images refer to the mixture of bacteria cells and nanomaterials, after 60 min of exposition. Figure 5a,a’ show the TEM and SEM micrographs of *Oenococcus oeni* (DSM7008) cells (incubated with the silica nanospheres without stirring). No damages in the cell structure or leakage of cytoplasm was observed during this stage. The TEM images of bacteria exposed to moving silica nanostructures show the nanospheres driven into the walls of *O. oeni* (DSM7008) cells (Figure 5b). SEM images of the bacteria treated with nanospheres confirmed that silica nanostructures were located around *O. oeni* (DSM7008) cells (Figure 5b’). Both images show that silica nanomaterials (after stirring) caused extensive damage and deformation of the cell structure. TEM and SEM images of silica nanospheres were additionally set with *O. oeni* (DSM7008) cells after treatment for the better identification of nanomaterials.

**Figure 6:**
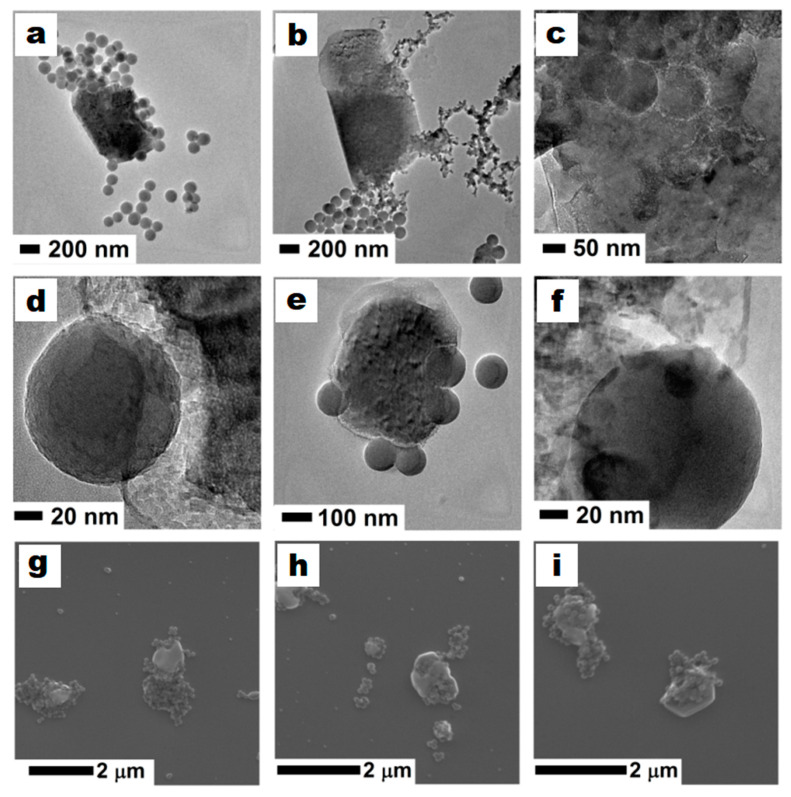
TEM (upper and middle panel) and SEM (lower panel) images of the *Oenococcus oeni* (DSM7008) cells stirred with the silica nanospheres (after 60 min). *O. oeni* (DSM7008) cell after silica nanosphere impact (**a**), cell deformation and release of its cytoplasmic matter (**b**), silica nanospheres inside bacteria cell (**c**), silica spheres immersed in a cell’s surface (**d–f**), SEM confirmation of large bacteria deformations and silica nanospheres surrounding *O. oeni* cells (**g–i**).

High-resolution TEM images showed that the cytoplasmic matter was released from the cells, and that their shape was deformed at the points of silica nanosphere impact (Figure 6). Magnification set on the impact area showed that half of the sphere could be immersed in a cell’s surface (Figure 6d,f). The reference picture for the high magnification images is presented in Figure 6e. The brighter rings (lower density) around nanospheres immersed in cells (Figure 6c) suggests that nanospheres had a great influence on the structure of the cells at the impact area, causing elevated deformation and disintegration of the cells. The microscopic analysis also revealed that *O. oeni* (DSM7008) cells after silica nanosphere impact and cell deformation tends to release its entrails (Figure 6a,b). The images also confirmed the observed leakage of cytoplasmic matter (Figure 6d–f). Furthermore, SEM images additionally confirmed TEM observations on large *O. oeni* (DSM7008) deformations and silica nanosphere drugging inside cells (Figure 6g–i). Previous studies on the interaction of silica nanomaterials and bacteria have shown that silica nanospheres incubated with streptomycetes (without stirring) can be dissolved and then partially internalised inside cells [55]. The additional TEM images of the nanospheres internalised by spores from the bacteria cells are presented in the Supplementary Materials (Figure S2).

**Figure 7:**
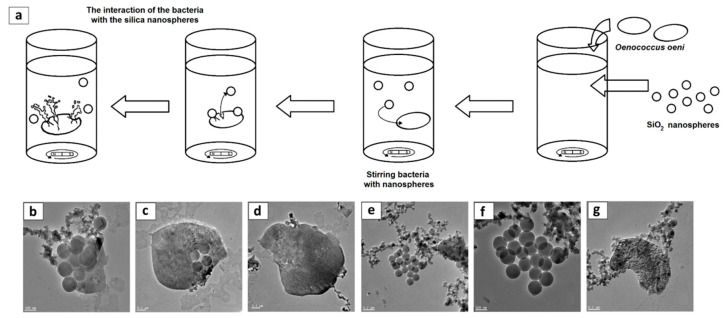
The diagram of *O. oeni* (DSM7008) cell disruption and release of cytoplasm caused by silica nanospheres (**a**) and TEM images of the “leaking” cells (**b**–**d**), as well as the absorption of the leakage by silica nanospheres (**e**–**g**).

The process of interaction of silica nanospheres with *O. oeni* (DSM7008) cells is presented in the diagram (Figure 7a). The transmission electron microscopy analysis proved that *O. oeni* (DSM7008) cells, after deformation by silica nanospheres, released cytoplasmic matter (Figure 7b–d) that is visible in the TEM images as a darker, long, worm-like structure. Further microscopic analysis showed that silica nanospheres tended to adsorb on the released cells’ interiors (Figure 7e–g).

### 3.6. Degradation of Silica Nanospheres

**Figure 8:**
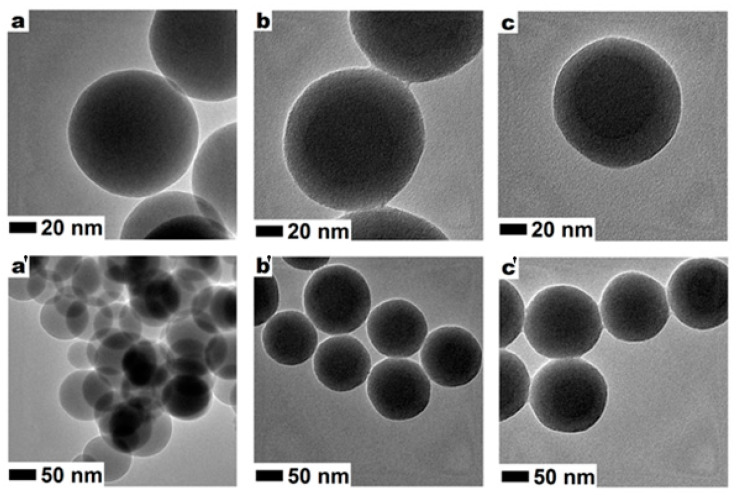
TEM images of the silica nanospheres in different magnifications before (**a**,**a’**) and after incubation in the glucose solution (**b**,**b’**) and PBS (**c**,**c’**).

The degradation of solid silica nanospheres were analysed with a transmission electron microscope (TEM) after the one-hour incubation in glucose and PBS solutions (without cells) at room temperature. A comparison of the images in Figure 8a–c indicates a partial/minor deformation of the tested nanospheres. TEM images in Figure 8b clearly indicate that the nanospheres incubated in the glucose solution started agglomerating with each other. However, no significant changes in diameter were recorded. Except for the observed agglomeration, changes in the shape of nanospheres were detected. The nanospheres exposed to the glucose solution have shifted from spherical towards oval or egg-shaped nanostructures. Furthermore, these nanospheres presented higher core/shell ratios, which is related to the detected changes in the density of the external and internal parts of the nanospheres. The comparison of the samples incubated in PBS buffer (Figure 8c) showed similar changes in the silica nanospheres. Comparable deformation was noticed when silica nanospheres were exposed to wine (Supplementary Materials, Figure S3).

## 4. Discussion

The stabilisation of wine without addition of SO_2_ poses a current challenge for the wine industry, therefore, winemakers and scientists are still looking for some alternative solutions. The possible application of nanomaterials in winemaking technology represents a recent approach in this field. Nanomaterials are interesting as antimicrobial agents for the degradation or removal of pollutants in wine or the immobilisation or vectorisation of yeast. To ensure microbial safety, silver-based nanomaterials have been studied for potential application in wine production. However, despite the great interest of their use as antimicrobial agents capable of eliminating or reducing sulphur dioxide in oenology, so far studies have been very scarce [56].

García-Ruiz et al. [57] studied silver nanoparticles (Ag NPs), stabilised with biocompatible materials, for controlling the growth of lactic acid bacteria (LAB) and acetic acid bacteria (AAB). The Ag NPs have been synthesised using biocompatible polyethylene glycol (PEG-Ag NPs (20.01% Ag content)) or glutathione (GSH-Ag NPs (0.197 mg/mL Ag content)). Authors have concluded that PEG-Ag NPs are more efficient against *E. coli* and AAB, while GSH-Ag NPs are highly efficient against *O. oeni*. Epifluorescence microscopy suggested damage to the integrity of the membrane after the incubation of wine bacteria with Ag NPs. Using the same nanomaterials and SO_2_ in different combinations, Gil-Sánchez et al. [58] tested their antimicrobial activity in white and red wine samples. Young white wine samples were collected before the addition of SO_2_ and silver nanoparticles (control), and after storage at 1 and 30 days for yeast, LAB, and AAB colony counting. Samples of finished red wine contaminated with a strain of *Brettanomyces bruxellensis* were taken at 15, 30, and 60 day of storage. The authors’ results showed the great potential of Ag NPs as antimicrobials to control LAB, AAB, and yeasts after alcoholic fermentation in wine, even more effectively than SO_2_. Tested materials also exhibited effectiveness against fastidious *B. bruxellensis* at low concentrations, but further research is needed about this ability during winemaking. Additional data indicated that the size and shape of the nanoparticles were almost unaltered in the case of GSH-Ag NPs, while in PEG-Ag NPs some particle agglomerations were observed. Furthermore, the authors’ results have suggested that Ag NPs may reach the intestine in a nano-scaled form; lastly, Caco-2 cell experiments seemed to exclude toxicity of Ag NPs at the intestinal epithelium. In contradiction to Gil-Sánchez et al. [58], our approach aimed at the short-term use of the silica nanomaterials. The possible application of the studied method (the use of silica nanospheres and stirring), may be performed as the post-fermentation treatment in winemaking technology, in a steel wine tank equipped with mixer. This method might serve as an opposition to the use of SO_2_ as wine additive, although more detailed study regarding enzymatic and chemical oxidation of wine is necessary.

The mechanism underlying the inhibitory effect of nanomaterials on microorganisms is so far unclear; however, it seems to have something in common with the difference in charge between the nanoparticles (positively charged) and the microbial cells (negatively charged) [59]. Microscopic analyses performed in our study showed that silica nanomaterials under stirring caused extensive damage (deformation) to the cell structure of *O. oeni* (DSM7008). The TEM images demonstrated the nanospheres driven into the walls of *O. oeni* (DSM7008) cells, whereas SEM images proved that silica nanostructures are located around bacteria, which supports the charge statement. Nanospheres at impact with the bacteria cell have a great influence on their structure, causing a large amount of the deformation and their disintegration. Therefore, the most probable mechanism causing over 80% decrease in the bacterial counts of *O. oeni* (Figure 2) is based on mechanical damage caused by an impact generated by the moving, charged silica nanostructures supported by the physiochemical interaction with cells. This notion was supported by the outcome of the cytotoxicity test. The performance of silica on *O. oeni* cells was present even without stirring (Figure 3), although the effect was considerably lower than in the case of stirred suspension. Furthermore, more time (3 h) was necessary to achieve a similar outcome. The fact that the effect was present even without stirring suggests that a negative charge displayed on the surface of nanostructures could play a role. Therefore, the increased effect in the stirred suspension should be accounted for, either from mechanical interactions or the higher availability of nanomaterial to the bacterial cells. As a result, the charge difference, combined with mechanical damages caused by the stirred silica spheres, could shorten the neutralisation time (90 min) and increase the efficiency of this process.

To our knowledge, any further research has described the inhibitory effect of nanomaterials on wine microorganisms. Therefore, with this paper, we present the first study on the use of silica nanospheres in control of *O. oeni* (DSM7008) numbers in liquid environments, which constitutes a novel approach in winemaking production, with the patent number PL 232150 B1 [60]. Due to the above, we must refer to related studies, which are focused on nanotechnology contributions in plant protection against pathogens; in the production of packages to ensure microbial safety of fresh and processed fruits and vegetables, including juices, as well as other liquids; and widely studied water purification.

Hashim et al. [61] tested ecofriendly nanomaterials, i.e., silica, chitosan, and copper nanoparticles (NPs), as well as their combination, for controlling grey mould of table grapes caused by *Botrytis cinerea*. Authors concluded that only one application of chitosan or silica NPs was able to reduce grey mould. Emamifar et al. [62] studied the effect of nanocomposite packaging on inactivation of *Lactobacillus plantarum* in orange juice. Film-grade LDPE (low density polyethylene) resin pellets were supported by antimicrobial agents, including P105 powder (TiO_2_ 95% and nanosilver 5% with particle diameters ~10 nm) and ZnO nanoparticles powder (~70 nm). In conclusion, they found more pronounced antimicrobial effects under the film containing nanosilver, especially at its higher content. In contrast to their research, our studies show that silica nanomaterial can be used alone for control of microbiological growth, instead of particles heaving bactericidal properties (e.g., TiO_2_ and ZnO). Similar to our studies, Youssef et al. [63] studied chitosan- and silica-based nanoparticles and suggested chitosan–silica nanocomposites as an alternative control means, in order to reduce or substitute the use of fungicides against the grey mould of table grapes. With regard to the Hashim et al. [61] and Youssef et al. [63] reports and our unpublished data, silica and silica-based nanomaterials did not alter the quality parameters of grapes and must fermentation, respectively. Therefore, nanosilica pose promising properties in microbial safety of fruits and their processing. Moreover, according to European Union (EU) regulation, silicon dioxide (E551) is an accepted food additive, in particular as an anti-caking agent [64]. In addition, the OIV (International Organisation of Vine and Wine), an intergovernmental organisation composed from scientific and technical entities with recognised competence for work concerning vines and wine, indicate silicon dioxide as one of the products that can be used to clarify wines [65]. This statement underlines the necessity to investigate the mode of action of this substance in the vinification process, and for that reason silica nanospheres were chosen in our study, as a potentially safe alternative approach to the microbial stabilisation of wine.

The cytotoxicity of silica nanoparticles seems to be dependent on the medium composition (PBS, 1% glucose solution, and young wine). Silica spheres maintained their activity in the wine environment and reduced the number of detected colonies by over 50% (Figure 4). This value was in between the recorded reduction in bacterial counts, reaching 82% and 38% for the PBS and glucose solutions, respectively (Figure 2). Therefore, conducted studies have shown that silica nanoparticles have the potential to be used in reducing the amounts of *O. oeni* in wine and other liquids. However, the nanomaterials have shown signs of mild deformation and started agglomerating (Figure 7; Supplementary Materials, Figure S3). A previous study on the stability of the silica nanostructures showed that trace amounts of sodium hydroxide and sodium chloride can influence the structure of silica nanomaterials [36]. In the case of mesoporous silica flakes, in the first step mesoporous structure degraded and additional cavities were formed. Furthermore, these cavities grew and additional silica structures started to appear. Finally, the flakes fell apart and a new shapeless structure took their place. With the core-shell mesoporous silica, nanosphere degradation occurred in similar steps. In the first step, the mesoporous structure degraded. Then the silica nanospheres started to melt one with another, and additional structures started to appear from fusing the remaining spheres of new spheres from dissolved silica [55]. These data confirm our observation of silica sphere degradation.

However, it should to be noted that in our study, the effect was tested on one *O. oeni* strain; therefore, further investigation on other LAB bacteria (including whole consortia) is necessary to verify if the effect can be up-scaled from the laboratory scale to operational environment. Further studies could be conducted in three directions, focusing on (i) malolactic consortium, (ii) biogenic amines producers, and (iii) spoilage organisms. This can be achieved in different stages of the vinification process.

The achieved results create a space for further testing of these materials in biotechnological processes, such as wine production. The described solution can be potentially used with other methods that remove bacteria from the process environment, which in wine production may lead to the reduction of SO_2_ used in the production.

## 5. Patents

Patent number PL 232150 B1, https://api-ewyszukiwarka.pue.uprp.gov.pl/api/collection/08d0a91f424ba7e8a4c32e3d8f1dcde0.

## Supplementary Materials

The following are available online at https://www.mdpi.com/2304-8158/9/9/1338/s1. Figure S1: Thermogravimetric analysis of silica nanospheres; Figure S2: TEM images of *Streptomyces* cell before (a), during (b), and after interaction with silica nanostructures (c), Figure S3: TEM images of pristine silica nanospheres (a) and after exposition in wine (b).
