# Supplementary material for: Potential Use of Silica Nanoparticles for the Microbial Stabilisation of Wine: An In Vitro Study Using Oenococcus oeni as a Model
Source: Foods. 2020 Sep 22;9(9):1338. doi: 10.3390/foods9091338 (PMC7555740; doi:10.3390/foods9091338)
Supplement: Supplementary file 1 [file foods-09-01338-s001.doc]

Potential use of silica nanoparticles in neutralisation of *O. oeni* in wine and liquids

Kamila Pachnowska ^1^, Krzysztof Cendrowski ^1^, Xymena Stachurska ^2^,*, Paweł Nawrotek ^2^, Adrian Augustyniak^3, 4^, Ewa Mijowska ^1^

^1^ Department of Nanomaterials Physicochemistry, Institute of Chemical and Environment Engineering, West Pomeranian University of Technology in Szczecin, Piastów Avenue 45, Szczecin 70-311, Poland; kamila.mijowska@zut.edu.pl (K.P.); krzysztof.cendrowski@zut.edu.pl (K.C.); ewa.mijowska@zut.edu.pl (E.M.)

^2^ Department of Microbiology and Biotechnology, Faculty of Biotechnology and Animal Husbandry, West Pomeranian University of Technology, Szczecin, Piastów Avenue 45, 70-311 Szczecin, Poland; pawel.nawrotek@zut.edu.pl

^3^ Department of Chemical and Process Engineering, Faculty of Chemical Technology and Engineering, West Pomeranian University of Technology, Szczecin, Piastów Avenue 42, 71-065 Szczecin, Poland; adrian.augustyniak@zut.edu.pl

^4^ Chair of Building Materials and Construction Chemistry, Technische Universität Berlin, Gustav-Meyer-Allee 25, 13355 Berlin, Germany

***** Correspondence: xymena.stachurska@zut.edu.pl

**Supporting information**





**Figure S1.** Thermogravimetric analysis of silica nanospheres.


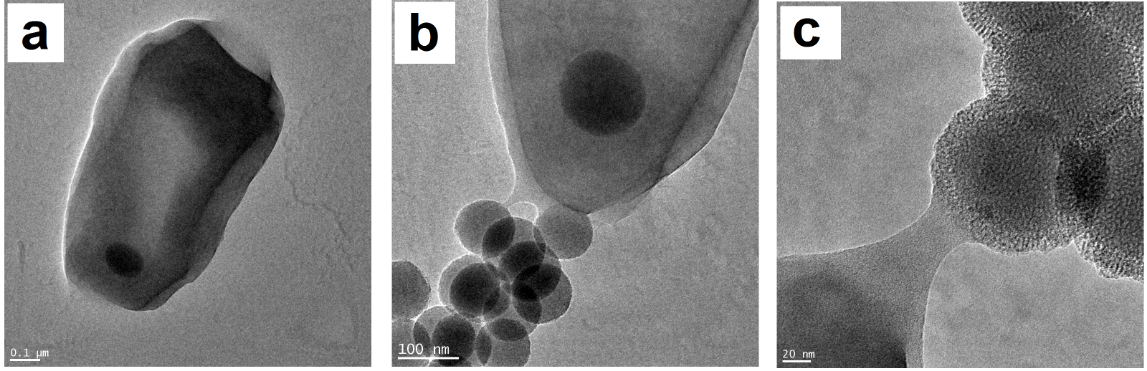


**Figure S2.** TEM images of *Streptomyces* cell before (**a**), during (**b**), and after interaction with silica nanostructures (**c**).


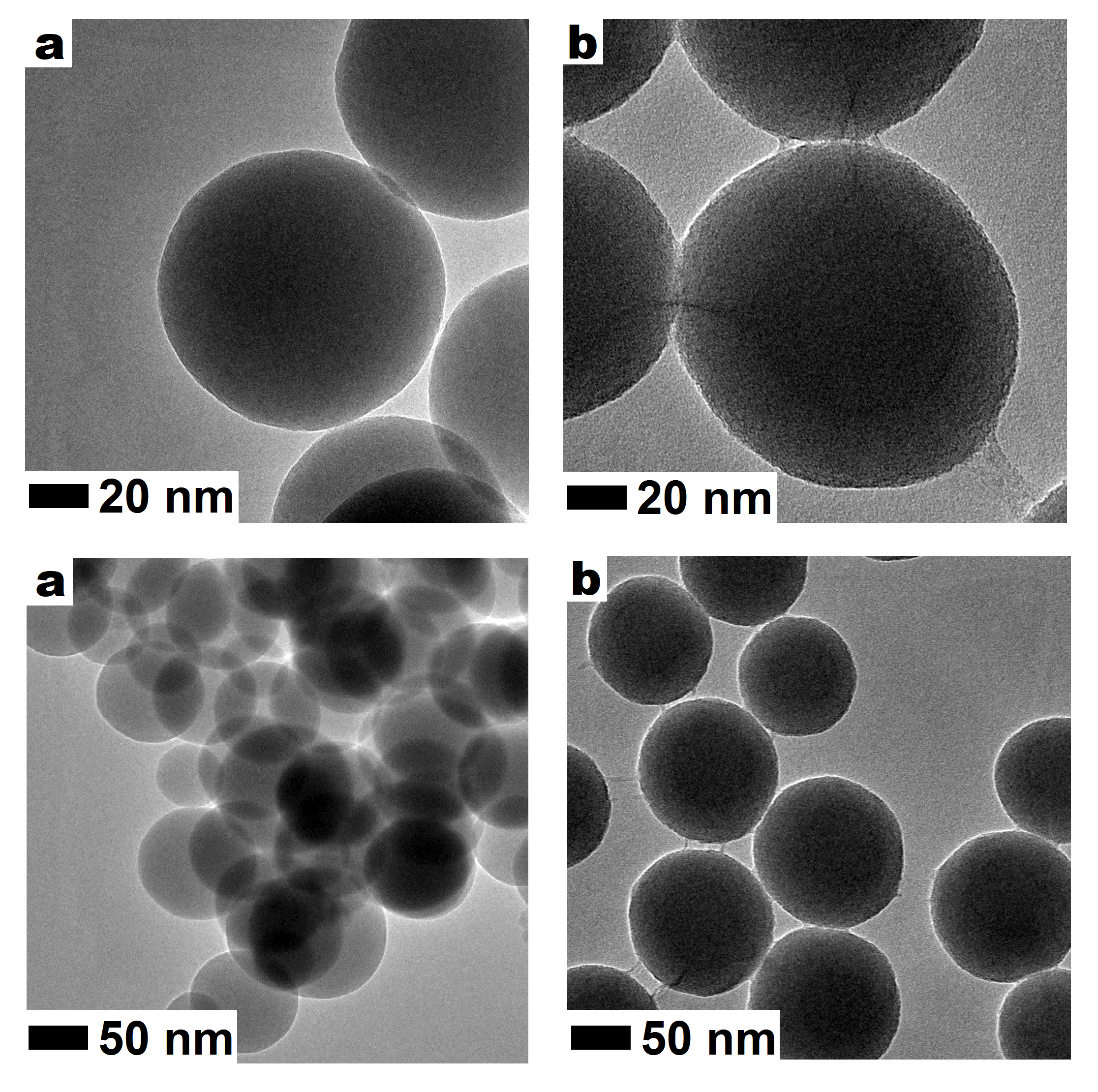


**Figure S3.** TEM images of pristine silica nanospheres (**a**) and after exposition in wine (**b**).
